# Experimental Study on the Fire Performance of Tubular Steel Columns with Membrane Protections for Prefabricated and Modular Steel Construction

**DOI:** 10.3390/ma11030437

**Published:** 2018-03-16

**Authors:** Xin Zhang, Lei Peng, Zhao-peng Ni, Tian-xiao Ni, Yi-liang Huang, Yang Zhou

**Affiliations:** 1 School of Civil Engineering, Central South University, Changsha 410075, China; zxcsu119@sina.com; 2 Changsha Kerich Fire Protection Engineering Tech. CO., Ltd., Changsha 410007, China; nitianxiao@sina.com; 3 Tianjin Fire Research Institute of the Ministry of Public Security, Tianjin 300381, China; penglei@tfri.com.cn (L.P.); nzhp_119@163.com (Z.N.); hyltfri@163.com (Y.H.)

**Keywords:** hollow structural section (HSS), steel tubular column, axial load, prefabricated and modular construction, membrane protection

## Abstract

Experimental research was conducted to study the fire resistance of steel tubular columns used in prefabricated and modular construction. In order to achieve high-efficient prefabrication and fast on-site installation, membrane protections using board products and thermal insulation blankets are adopted as the favorable protection method. Three protected tubular columns were tested in a full-scale column furnace with axial load applied. The study variables were different membranes, including fiber reinforced calcium silicate (FRCS) boards, rock wool and aluminum silica (Fiberfrax) insulations. The results suggest that one layer of 12 mm FRCS board with rock wool insulation has insufficient fire protection. However, steel columns protected with two layers of 12 mm FRCS boards with insulation appeared to have good fire resistances and could achieve a fire resistance rating as high as 2.5~3.0 h.

## 1. Introduction

Prefabricated and modular steel structures are gaining more and more popularity in China recently. By prefabricating modular sections or components that are assembled to complete construction, project time and cost can be significantly reduced [1,2,3].

A 30-storey hotel building was completed in just 15 days using prefabricated and modular construction technology in China [4]. The hotel was assembled using modular steel columns, floor assemblies and light-frame walls. It was reported that this high-efficient construction mode takes only 7% of the total construction hours and 20% of the energy consumption when compared with the conventional construction mode and it saves 10~30% of the construction costs [5].

The Chinese code requires that columns in high-rise buildings have a fire resistance rating of either 2.5 h or 3.0 h, depending on the type of the building [6]. To meet the requirements, steel columns in the prefabricated and modular construction must be protected [7,8,9,10,11,12,13]. However, the fire protection must be optimized to fit the process of prefabrication in factory and modular assembly on-site.

Generally, there are various methods of protection to reduce the rate of heat transfer to structural steel and the most popular methods include the use of spray-applied materials, concrete encasement and board products and so forth [14,15,16,17,18,19,20]. In order to achieve factory-made efficiency and optimize the on-site installation process, spray-applied materials and concrete encasement on structural steel are not favorable methods of protection for prefabricated and modular technology.

Using board products as membrane protection, fire rated gypsum board is the most commonly used material to protect structural steel. However, to achieve a fire resistance of 2.5 h or 3.0 h, the thickness and number of layers of gypsum boards must be considerably increased and this does not favor the factory process at all. Therefore, a board product, namely fiber reinforced calcium silicate (FRCS) board, is considered as an option for the fire protection of steel columns in prefabricated and modular construction. The FRCS is manufactured from siliceous and calcareous materials reinforced with fibers and autoclaved to produce a stable crystalline structure. The stable crystalline structure and autoclaving makes the board very strong, durable and stable at high temperatures.

The fire performance of the tubular steel column with protections has attracted more and more attention in recent years. Mariappan [16] reviewed some recent work related to the development of an intumescent fire protective coating, which was suitable for the protection of non-flammable substrates, such as structural steel, against fire. Alam et al. [21] investigated the later load resistance of non-insulated steel columns under fire exposure using finite element analysis. Torić et al. [8] presented a newly developed numerical model for the behavior of steel structures exposed to fire, capable of taking into account the effect of steel creep at high temperatures by using an implicit creep model, as well as the experimental verification of the model. Yang and Yu [10] tested a series of fire-resistant steel columns with three different slenderness ratios under a sustained load under a uniform temperature for up to six hours in order to evaluate the creep upon three selected factors—temperature, applied load and column slenderness. Choe et al. [12] presented the behavior of axially loaded steel columns subjected to thermal gradients through their cross sections. Chen et al. [15] conducted twenty tests to investigate the efficiency of the intumescent coating designed to protect steel plate at an elevated temperature, by means of electrical furnace. Agarwal et al. [13] evaluated the behavior and design of steel columns subjected to thermal gradients due to fire loading numerically and experimentally. Liu et al. [22] report a full-scale fire testing of a concrete filled thin-walled steel tubular (CFST) column-wall structure.

This research was intended to study the fire resistance of steel tubular columns used in prefabricated and modular construction. Three tubular columns with different membrane protections were tested in a full-scale column furnace to study the fire resistance of columns. This paper concludes appropriate fire protection methods for the steel tubular columns to meet the code requirements. The test results provide necessary knowledge for the fire resistance design of steel columns used in prefabricated modular construction.

## 2. Materials and Methods 

### 2.1. Specimens and Instrumentation

**Figure 1:**
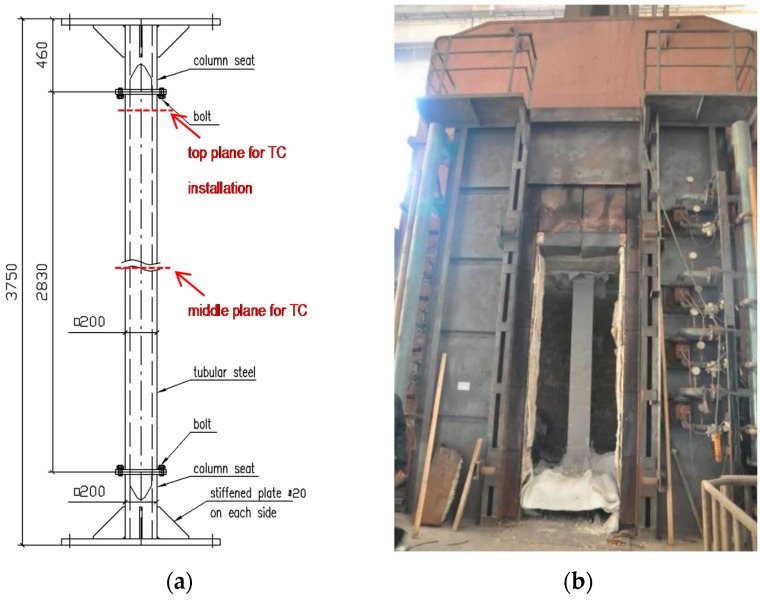
(**a**) Design of the column; (**b**) a protected column mounted within the furnace.

Three specimens were prepared for the fire resistance tests. The specimens are tubular columns, representing typical columns used in prefabricated and modular construction. The columns have hollow structural sections (HSS) in a square shape of 200 × 200 mm, with different steel thicknesses of 10, 20 and 30 mm. A specimen has a net section height of 2830 mm, connecting to column seats at each end with 16 bolts. The total height of each specimen is 3750 mm. The column details are shown in Table 1 and Figure 1.

The columns were made of Q345B steel, of which the steel yield strength is 345 MPa. The column design strengths are given in Table 1, calculated based on the Chinese steel structure standard [23].

The three specimens were protected using different materials, as shown in Table 1. For HSS-C1, the protection materials were rock wool fiber and one layer of FRCS board; for HSS-C2, rock wool fiber and two layers of FRCS boards were used; for HSS-C3, the steel column was wrapped up with 25 mm thick Fiberfrax and then protected with rock wool fiber and two layers of FRCS boards. To install the protections, C-shape light gauge steel studs (50 × 50 × 0.7 mm) were used to attach FRCS boards. Rock wool fibers were filled up in cavities between steel studs. The FRCS boards had an average density of 950 kg/m^2^ and the thermal conductivity of 0.15 W/(m·K) at 20 °C. The rupture strength of FRCS boards was 6.0 MPa. The Fiberfrax blanket products are manufactured from alumina-silica materials and exhibit thermal stability at high temperatures with low thermal conductivity. Rock wool is made from molten volcanic rock (typically basalt or dolomite), spun into a fiber-like structure, which provides a high-quality of insulation.

**Figure 2:**
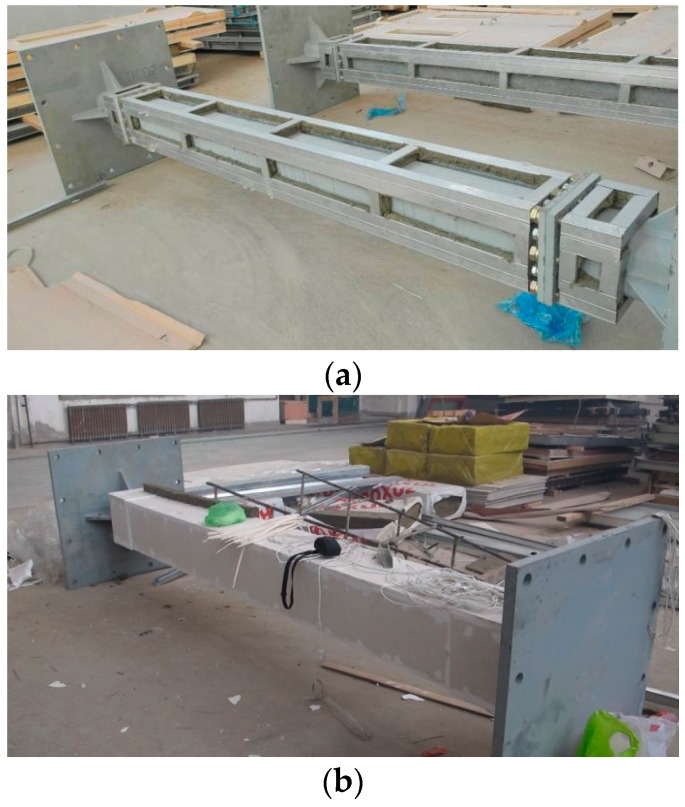
Fabrication of the column protection: (**a**) unprotected; (**b**) protected.

**Figure 3:**
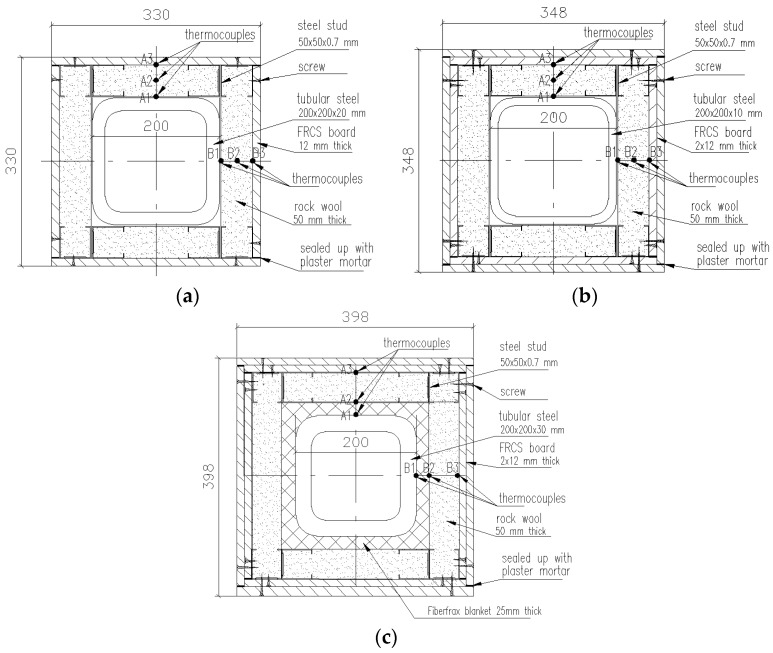
Cross-section view of the three columns with protection: (**a**) HSS-C1; (**b**) HSS-C2; (**c**) HSS-C3.

Figure 2 shows fabrication of the column protection. The column protection details are shown in Figure 3 in a cross-section view.

For each column specimen, twelve thermocouples were installed on two planes to measure specimen temperatures at different locations. Six of them were located in a plane at the middle height of the column (marked as M) and the other six were located in a plane at the top (marked as T), shown in Figure 1. On each plane, thermocouple A1 (B1) represents steel temperatures; thermocouple A2 (B2) represents temperatures in the middle of the insulation; A3 (B3) represents temperatures between the FRSC board and insulation. Figure 3 shows the thermocouple locations on each plane. The gaps between FRCS boards were sealed up with a mortar, which was made from mixed water-glass (sodium silicate) and refractory cement. This mortar exhibited good performance at high temperatures and stayed in place as observed during the tests.

Liner variable displacement transducers (LVDTs) were also installed to measure the axial contraction of the columns.

In order to avoid damage of columns during transportation, precautions should be made. Cushions were used to prevent any possible collision of prefabricated columns during transportation.

### 2.2. Test Methodology

The column tests were conducted using a full-scale column furnace, shown in Figure 1. The furnace has interior dimensions of 3000 × 3000 × 3000 mm (Width × Height × Depth). The loading system is capable of applying a 15,000 kN compressive axial load.

The furnace temperatures can be measured and controlled by 9 K-Type thermocouples. The average time-temperature curve followed the Chinese standard curve (GB/T 9978.1 [24]). The curve is equivalent to the ISO 834-1 curve [25].

Failure of the column is deemed to occur if the axial contraction exceeds h/100 (mm), or the rate of axial contraction exceeds 3 h/1000 (mm/min), where h (mm) is the initial height of the column.

### 2.3. Test Procedures

For each test, the specimen was installed in the furnace and both ends were hinged. Prior to the test, an axial load was applied to the specimen at 66% of the design strength for 30 min. The specimen was then exposed to standard fire generated in the furnace. The load was maintained constantly during the test.

If the failure criteria were met, the test ended. After cooling down, the specimen was removed from the furnace for inspection.

## 3. Results

### 3.1. Observations

**Figure 4:**
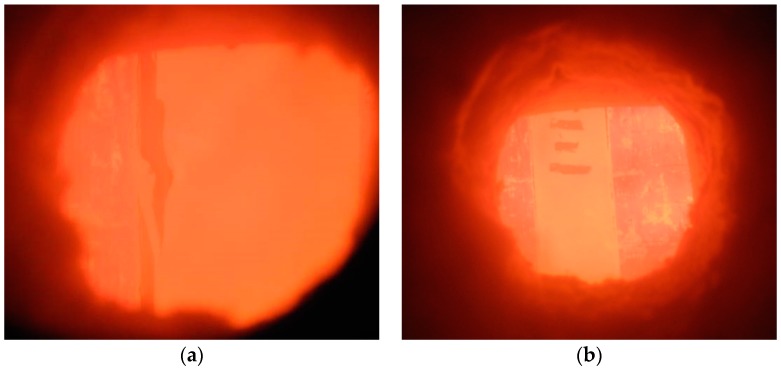
View of test specimens through the observation window: (**a**) HSS-C1; (**b**) HSS-C3.

For HSS-C1, after about 10 min, the board joints started to open up slightly. At about 20 min, the board joints opened up significantly, shown in Figure 4a. The steel studs and rock wool were then exposed to fire exposure at the opened joints. At 74 min, the column lost its strength and failed.

**Figure 5:**
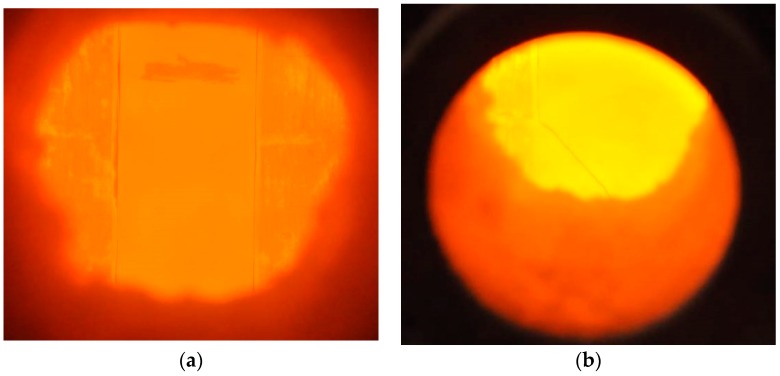
View of test specimen HSS-C2 through the observation window: (**a**) at about 10 min; (**b**) at about 25 min.

For HSS-C2, after about 10 min, the board joints opened up slightly, shown in Figure 5a. The sealing mortar started to fail off. At about 25 min, a crack appeared at the bottom left on the front side of the FRCS board but did not fall off during the rest of the test, shown in Figure 5b. At 180 min, the column failed.

For HSS-C3, after about 10 min, the board joints started to open up slightly. No cracks appeared on the boards during the 3.0 h tests, shown in Figure 4b. The fire resistance test was terminated at 180 min with safety concerns, because the furnace was designed to run fire tests within 3.0 h. The column did not show any sign of failure at the end of the test. Therefore, the ultimate fire resistance of the column of HSS-C3 would be larger than 3.0 h.

**Figure 6:**
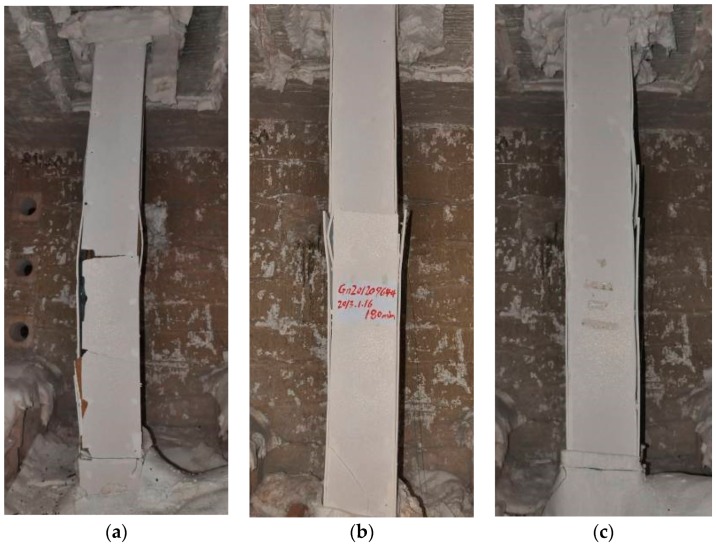
Columns after tests (still in the furnace): (**a**) HSS-C1; (**b**) HSS-C2; (**c**) HSS-C3.

The results of the three full-scale fire resistance tests are summarized in Table 2. The three columns after fire tests are shown in Figure 6. Note that the FRCS boards for specimen HSS-C3 did not break during the test. However, the breaks occurred during the cooling time.

**Figure 7:**
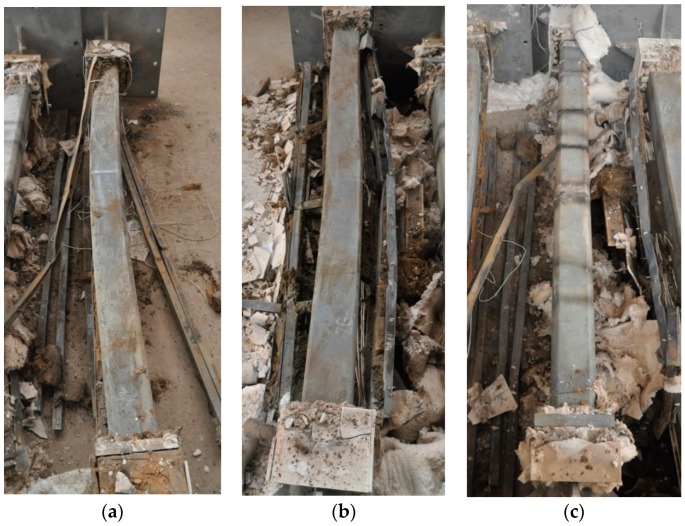
Columns after tests (protections removed): (**a**) HSS-C1; (**b**) HSS-C2; (**c**) HSS-C3.

Figure 7 shows the three columns with protection removed. Both specimen HSS-C1 and HSS-C2 show considerable deformation of buckling at about the middle height of the columns, while the specimen HSS-C3 shows no apparent deformation.

### 3.2. Measurements

**Figure 8:**
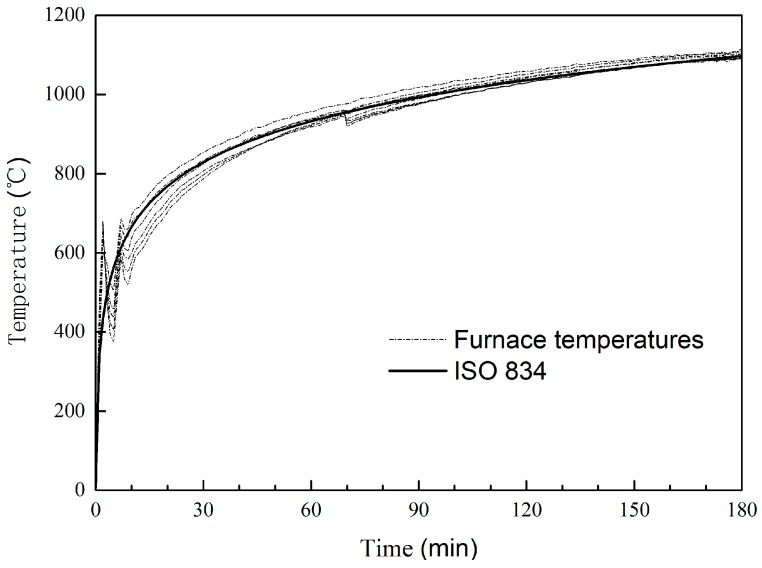
Furnace temperatures for HSS-C2.

The measured furnace temperatures for specimen HSS-C2 are plotted in Figure 8 as an example. During the first 10 min, the temperatures had some oscillation. After 10 min, the average furnace temperature followed the standard temperature curve very well.

**Figure 9:**
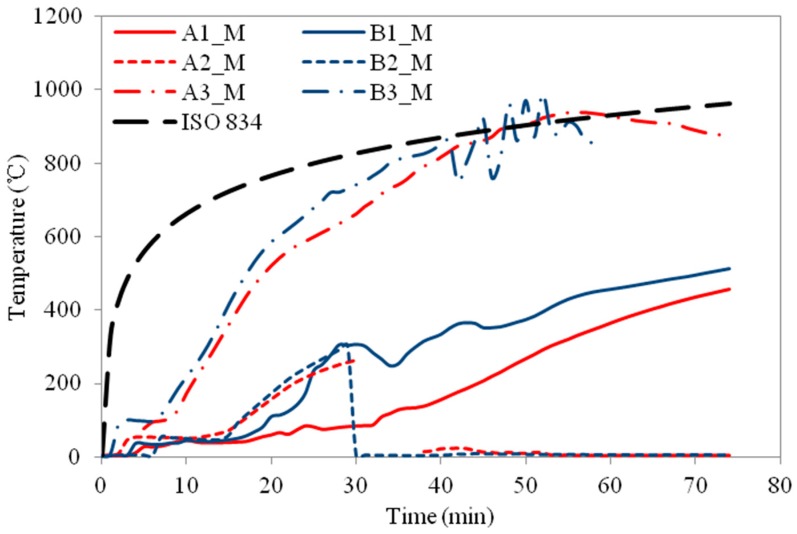
Temperature profiles at the medium section for HSS-C1.

**Figure 10:**
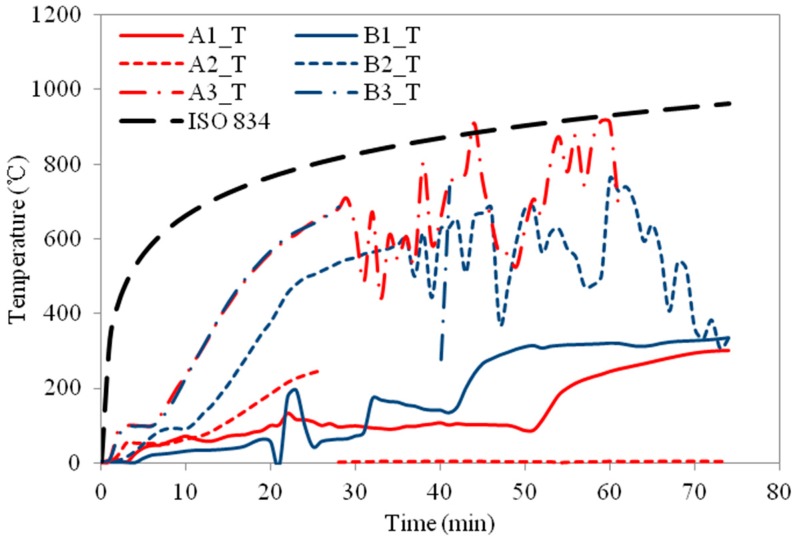
Temperature profiles at the top section for HSS-C1.

**Figure 11:**
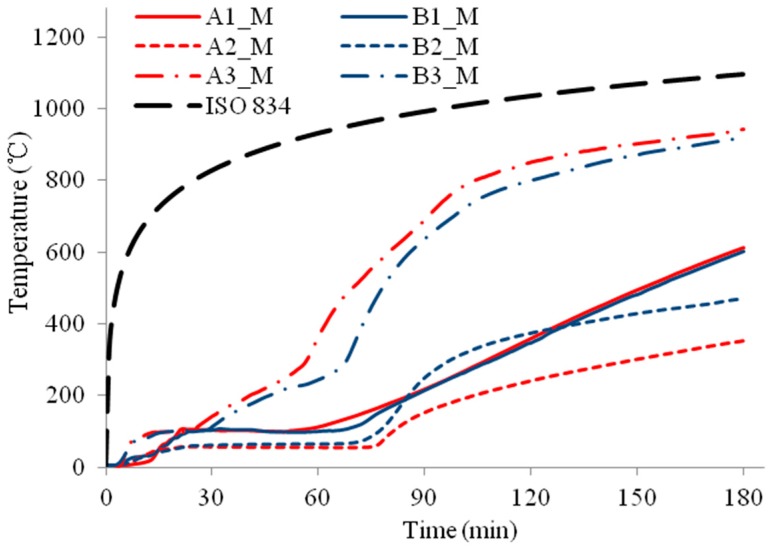
Temperature profiles at the medium section for HSS-C2.

**Figure 12:**
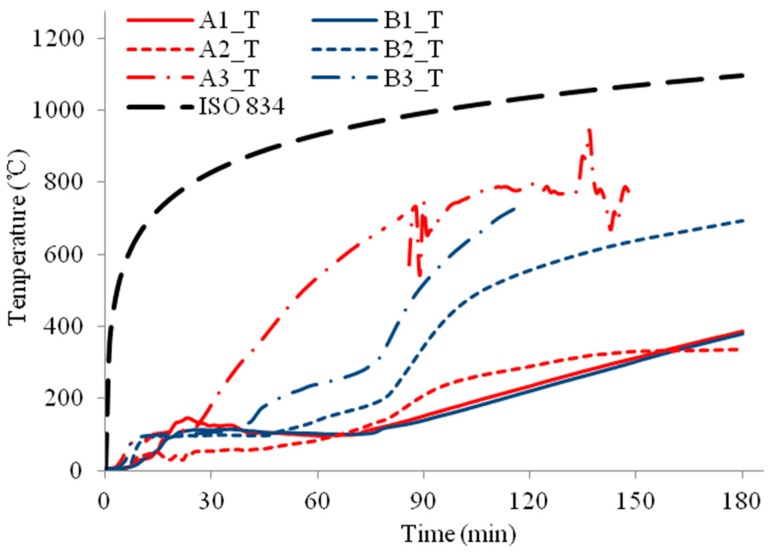
Temperature profiles at the top section for HSS-C2.

**Figure 13:**
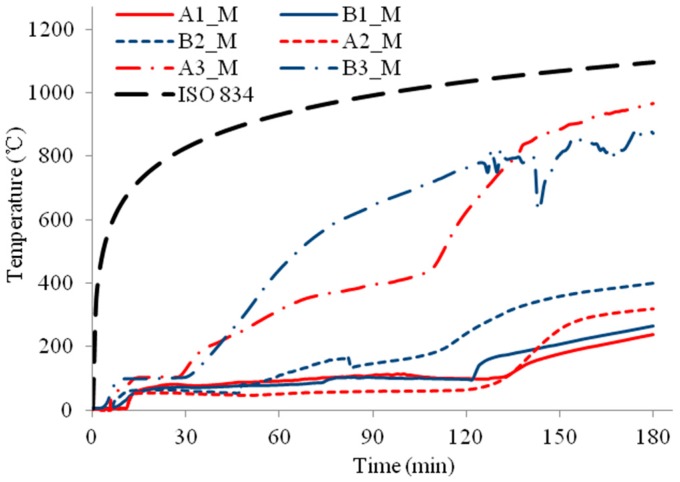
Temperature profiles at the medium section for HSS-C3.

**Figure 14:**
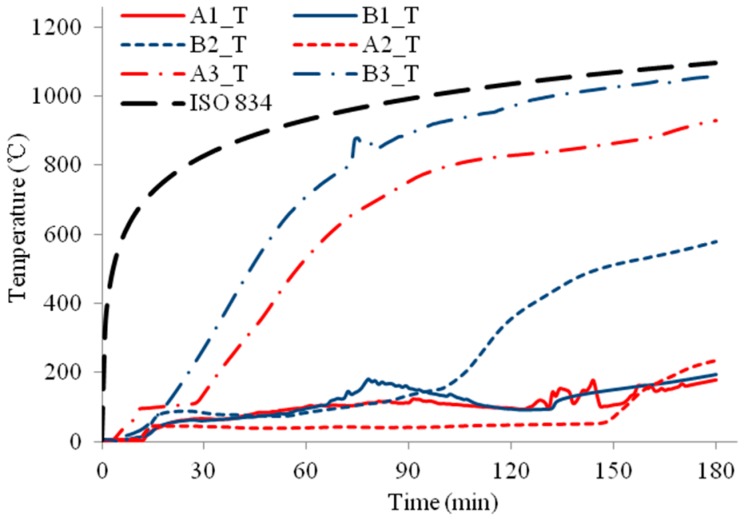
Temperature profiles at the top section for HSS-C3.

**Figure 15:**
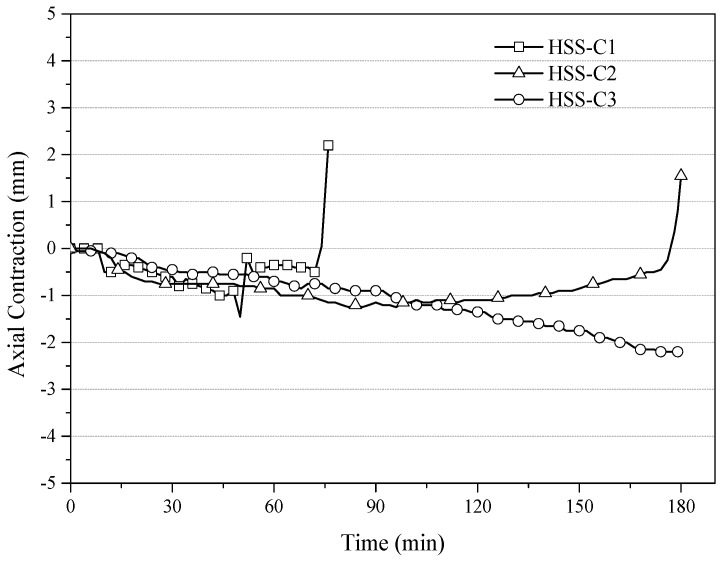
Axial contractions of the three columns.

The temperature profiles of the three column specimens are shown in Figure 9, Figure 10, Figure 11, Figure 12, Figure 13 and Figure 14 and the measured axial contractions of the three columns are shown in Figure 15.

For specimen HSS-C1 protected with one layer of FRSC board, the temperatures behind the boards (A3 and B3) started to increase rapidly after about 7~8 min exposure (Figure 9 and Figure 10). At about 45 min, the A3 and B3 temperatures reached the same as the furnace temperature (Figure 9). This indicated that the thermocouples were exposed to direct fire exposure. At 74 min when the column failed, the steel temperature at the middle plan (B1_M) reached 500 °C. Figure 7 shows that the column experienced considerable deformation in the test.

For specimens HSS-C2 and HSS-C3 protected with a double-layer of FRSC boards, temperatures between the boards and insulation (A3 and B3) had similar increase trends, as shown in Figure 11 and Figure 13. At 180 min, the furnace temperature was about 1100 °C and the A3 and B3 temperatures reached about 900~1000 °C (Figure 11, Figure 12 and Figure 13), which were less than the furnace temperature. This indicated that the boards did not break before 180 min and the rock wool did not experience direct exposure to the fire during the tests.

For specimen HSS-C2, the column steel temperatures (A1 and B1) increased slowly before 60 min, as shown in Figure 11 and Figure 12. After about 60~70 min, the column temperatures started to increase almost linearly. At 180 min, the temperatures at the middle height of the column (A1_M and B1_M) reached about 600 °C. Accordingly, it can be observed in Figure 7 that the column experienced considerable deformation at these high temperatures.

For HSS-C3, the steel column was wrapped up with Fiberfrax blanket. This insulation cut out the heat transfer from the steel studs to the column and provided excellent insulation for the steel column. As shown in Figure 13 and Figure 14, at the end of the test, the column temperatures were about 200~250 °C, which is much less than the critical temperature of 550 °C for structural steel. The column did not fail at 3.0 h but the test had to be terminated due to the design safety of the furnace. It can be confirmed that the column experienced no visible deformation in Figure 7.

For HSS-C1, the temperature measurements at some locations show considerable variance and disturbance (e.g., A2_M and B2_M, A2_M and A2_T, etc.). This is because that HSS-C1 was the first test specimen and there was some compatibility problem between the instrumentations and acquisition system. The variance and disturbance were minimized in tests HSS-C2 and HSS-C3 by solving the measuring problem.

The measured axial contractions are shown in Figure 15. The negative values represent that the columns were experiencing thermal expansion. For HSS-C3, the specimen underwent thermal expanding continuously throughout the test. However, HSS-C1 and HSS-C2 experienced a fast increase in the axial displacement near the end of the tests. The columns failed due to the criterion of the rate of axial contraction.

### 3.3. Discussion

The steel tubular column, protected with one layer of 12 mm FRCS board and 50 mm rock wool, had a fire resistance rating of 74 min. The steel furring studs experienced large deformation at high temperatures and this resulted in the opening-up of the FRCS boards and reduced the fire protection effect of the boards and rock wools.

The tubular column, protected with two layers of 12 mm FRCS board and 50 mm rock wool, had a fire resistance rating of 180 min. The FRCS boards were maintained in place during the test and the integrity provided protection for the rock wool and the column. At 180 min, the column temperatures reached 600 °C and the column failed. It indicated that this protection method could provide a fire resistance rating of 3.0 h with no safe margin. Nevertheless, this protection could be used to provide a fire resistance rating of 2.5 h with good safe margins.

Moreover, the tubular column, protected with 25 mm Fiberfrax and two layers of 12 mm FRCS board combined with 50 mm rock wool, experienced very good fire resistance performance during the 3.0 h test. At 180 min, the column temperatures reach only about 200~250 °C. Therefore, the protection is able to provide a fire resistance rating of 3.0 h with a good safe margin.

## 4. Conclusions

Based on the experimental work conducted in this research, we can conclude that appropriate fire protection methods are necessary for the steel tubular columns to meet the Chinese code requirements for the fire resistance rating. One layer of 12 mm FRCS board and 50 mm rock wool could not provide sufficient fire protection. Two layers of 12 mm FRCS board and 50 mm rock wool provided a fire resistance rating of no less than 2.5 h to steel tubular columns, while the tubular column, protected with 25 mm Fiberfrax and two layers of 12 mm FRCS board combined with 50 mm rock wool, could have a fire resistance rating of more than 3.0 h. In the tests, it was observed that the fire protection did not fall prematurely before the failure of the columns. It is worth mentioning that if the fire protection falls prematurely during the test, the high temperature intrusion will affect the columns’ load capacity and reduce the fire resistance rating accordingly.

In the future, the authors will carry out a further study on numerical modelling and a parametric study of the fire resistance of tubular steel columns, as well as the fire resistance of steel truss floor assemblies with the protection of FRCS boards and rock wool. This research project will provide necessary knowledge and benefit the fire safety development for prefabricated and modular steel construction.

## Figures and Tables

**Table 1:** Column specimens for fire resistance tests.

Specimen	Section Size (mm)	Net Section Height (mm)	Total Height (mm)	Column Strength (kN)	Protection
HSS-C1	200 × 200 × 20	2830	3750	3431	50 mm rock wool + 12 mm FRCS board
HSS-C2	200 × 200 × 10	2830	3750	1922	50 mm rock wool + 2 × 12 mm FRCS boards
HSS-C3	200 × 200 × 30	2830	3750	4766	25 mm Fiberfrax blanket + 50 mm rock wool + 2 × 12 mm FRCS boards

**Table 2:** Test results of the columns.

Specimen	Section Size (mm)	Protection Method	Column Strength (kN)	Load Applied (kN)	Fire Resistance (min)
HSS-C1	200 × 200 × 20	50 mm rock wool + 12 mm FRCS board	3431	2278	74
HSS-C2	200 × 200 × 10	50 mm rock wool + 2 × 12 mm FRCS boards	1922	1272	180
HSS-C3	200 × 200 × 30	25 mm Fiberfrax blanket + 50 mm rock wool + 2 × 12 mm FRCS boards	4766	3196	>180

## References

[1] Liu X., Xu A., Zhang A., Ni Z., Wang H., Wu L. (2015). Static and seismic experiment for welded joints in modularized prefabricated steel structure. J. Constr. Steel Res..

[2] Zhang A., Liu X. The new development of industrial assembly high-rise steel structure system in China. Proceedings of the Tenth Pacific Structural Steel Conference.

[3] Dao T., Lindt J. (2013). Seismic Performance of an Innovative Light-Frame Cold-Formed Steel Frame for Mid-Rise Construction. J. Struct. Eng..

[4] Broad Group Global Tall Building Database of the CTBUH, T30 Hotel [EB/OL]. http://skyscrapercenter.com/changsha/t30-tower-hotel/14432.

[5] Broad Group T30A Tower Hotel—Technical Briefing [EB/OL]. http://www.broad.com/Storage/Largedownloads/tsjd.pdf.

[6] GB 50016-2014 (2015). Code for Fire Protection Design of Buildings.

[7] Xue Y., Zhang S., Yang W. (2015). Influence of expanded vermiculite on fire protection of intumescent fireproof coatings for steel structures. J. Coat. Technol. Res..

[8] Torić N., Harapin A., Boko I. (2014). Experimental Verification of a Newly Developed Implicit Creep Model for Steel Structures Exposed to Fire. Eng. Struct..

[9] Wang J. (2016). The protective effects and aging process of the topcoat of intumescent fire-retardant coatings applied to steel structures. J. Coat. Technol. Res..

[10] Yang K., Yu Z. (2013). Experimental research on the creep buckling of fire-resistant steel columns at elevated temperature. Steel Compos. Struct..

[11] Morovat M., Engelhardt M., Helwig T. (2014). High-Temperature Creep Buckling Phenomenon of Steel Columns Subjected to Fire. J. Struct. Fire Eng..

[12] Choe L., Agarwal A., Varma A. (2016). Steel Columns Subjected to Thermal Gradients from Fire Loading: Experimental Evaluation. J. Struct. Eng..

[13] Agarwal A., Choe L., Varma A. (2014). Fire design of steel columns: Effects of thermal gradients. J. Constr. Steel Res..

[14] Milke J.A. (2016). Analytical Methods for Determining Fire Resistance of Steel Members. SPFE Handbook of Fire Protection Engineering.

[15] Chen C., Zeng J., Shen B. (2015). Experimental investigation on performance of intumescent coating for steel plate at elevated temperature. J. Cent. South Univ..

[16] Mariappan T. (2016). Recent developments of intumescent fire protection coatings for structural steel: A review. J. Fire Sci..

[17] Ullah S., Ahmad F., Shariff A. (2014). Synergistic effects of kaolin clay on intumescent fire retardant coating composition for fire protection of structural steel substrate. Polym. Degrad. Stab..

[18] Akaa O., Abu A., Spearpoint M. (2016). A group-AHP decision analysis for the selection of applied fire protection to steel structures. Fire Saf. J..

[19] Ullah S., Ahmad F., Yusoff P. (2013). Effect of boric acid and melamine on the intumescent fire-retardant coating composition for the fire protection of structural steel substrates. J. Appl. Polym. Sci..

[20] Uy B., Patel V., Li D., Aslani F. (2017). Behaviour and design of connections for demountable steel and composite structures. Structures.

[21] Alam M., Billah A., Quayyum S. (2013). Fire performance curves for unprotected HSS steel columns. Steel Compos. Struct..

[22] Liu J., Han L., Zhao X. (2017). Performance of concrete-filled steel tubular column-wall structure subjected to ISO-834 standard fire: Experimental study and FEA modelling. Thin-Walled Struct..

[23] GB 50017-2003 (2003). Code for Design of Steel Structures.

[24] GB/T 9978.1-2008 (2008). Fire-Resistance Tests—Elements of Building Construction—Part 1: General Requirements.

[25] ISO 834-1:1999 (1999). Fire-Resistance Tests—Elements of Building Construction—Part 1: General Requirements.

